# Parasitic diseases of camels in Iran (1931–2017) – a literature review

**DOI:** 10.1051/parasite/2017024

**Published:** 2017-06-15

**Authors:** Alireza Sazmand, Anja Joachim

**Affiliations:** 1 Zoonotic Diseases Research Center, School of Health, Shahid Sadoughi University of Medical Sciences Postal Code 8915173160 Yazd Iran; 2 Institute of Parasitology, Department of Pathobiology, University of Veterinary Medicine Vienna Veterinaerplatz 1 1210 Vienna Austria

**Keywords:** dromedary, Bactrian camel, *Camelus*, review, Iran

## Abstract

Parasitic diseases of camels are major causes of impaired milk and meat production, decreases in performance or even death. Some camel parasites also represent a threat to human health. About 171,500 one-humped camels (*Camelus dromedarius*) and 100–300 two-humped camels (*Camelus bactrianus*) live in Iran. Knowledge of the biodiversity of their parasites is still limited. The present review covers all information about camel parasitic diseases in Iran published as dissertations and in both Iranian and international journals from 1931 to February 2017. Ten genera of Protozoa (*Trypanosoma*, *Eimeria*, *Cryptosporidium*, *Toxoplasma*, *Neospora*, *Sarcocystis*, *Besnoitia*, *Theileria*, *Babesia* and *Balantidium*), 48 helminth species detected in the digestive system, including three species of Trematoda, four species of Cestoda, and 41 species of Nematoda, as well as helminths from other organs – *Echinococcus* spp., *Dictyocaulus filaria*, *Thelazia leesei*, *Dipetalonema evansi* and *Onchocerca fasciata* – have so far been described in Iranian camels. Furthermore, 13 species of hard ticks, mange mites, the myiasis flies *Cephalopina titillator* and *Wohlfahrtia magnifica*, and immature stages of the Pentastomida *Linguatula serrata* have also been reported from camels of Iran. Camel parasitic diseases are a major issue in Iran in terms of economics and public health. The present review offers information for an integrated control programme against economically relevant parasites of camels.

## Abstract (fr)

Les maladies parasitaires sont des causes majeures de diminution de production du lait et de la viande, diminution des performances ou même mort. Certains parasites de dromadaires représentent également une menace pour la santé humaine. Environ 171 500 dromadaires (*Camelus dromedarius*) et 100 à 300 chameaux à deux bosses (*Camelus bactrianus*) vivent en Iran. La connaissance de la biodiversité de leurs parasites est encore limitée. La présente revue couvre toutes les informations sur les maladies parasitaires des Camelidae en Iran qui ont été publiées dans des thèses et dans des revues iraniennes et internationales de 1931 à février 2017. Dix genres de Protozoaires (*Trypanosoma*, *Eimeria*, *Cryptosporidium*, *Toxoplasma*, *Neospora*, *Sarcocystis*, *Besnoitia*, *Theileria*, *Babesia* et *Balantidium*), 48 espèces d’helminthes détectées dans le système digestif, dont trois espèces de Trematoda, quatre espèces de Cestoda et 41 espèces de Nematoda, ainsi que des helminthes d’autres organes – *Echinococcus* spp., *Dictyocaulus filaria*, *Thelazia leesei*, *Dipetalonema evansi* et *Onchocerca fasciata* – ont jusqu’ici été décrits chez les Camelidae iraniens. En outre, 13 espèces de tiques, des acariens, les mouches à myiases *Cephalopina titillator* et *Wohlfahrtia magnifica*, et les stades immatures du Pentastomide *Linguatula serrata* ont également été signalés chez les Camelidae en Iran. Les maladies parasitaires des Camelidae doivent être considérées comme un problème en Iran en termes d’importance économique et de santé publique. La présente revue offre des informations pour un programme de contrôle intégré contre les parasites économiquement pertinents des Camelidae.

## Introduction

Camel medicine has a long history in Iran, and the book of Abu Obayda Mamar b. Motanna Bajarvani (died circa 827) on camels, namely *Ketaab al-ebel*, in English*, The book of dromedaries,* is among the earliest works about camels compiled by a Persian. This book on animal physiology and veterinary science was written in 16 chapters. The 9th century physician Ali b. Rabban Tabari described the functions of parts of the bodies of various animal species: camels, bulls, donkeys, elephants, and lions, in the fourth communication of his *Ferdaws al-ḥekma* (in English, *The paradise of wisdom*) (pp. 421–427), and discussed animal diseases and their appropriate treatments [154]. Traditional methods for controlling animal diseases were also described. Camels suffering from surra (*Trypanosoma* infection, known in Iran as *del zanak*) were firmly bound, and small rocks, heated up in a fire, were attached to a board that was moved under the neck of the animal. This type of cauterisation was believed to confer protection; it perhaps stimulates the immune system [164].

Modern veterinary knowledge found its way into Iran during the 1850s via European veterinarians who were mainly in charge of royal stables and military services [154]. However, the first records of veterinary parasitology activities in Iran are reports by Carpentier in 1931 who diagnosed *Trypanosoma evansi*-like trypomastigotes in the blood of sick horses in the south of Iran [26]. Since the 1930s, Iranian veterinarians have been trained in faculties of veterinary medicine in Iran and have conducted modern veterinary parasitology research. According to the latest official report, there are about 171,500 Old World Camels (OWCs) including one-humped (dromedary) and two-humped (Bactrian) camels (only 100–300 individuals) in Iran, which are scattered throughout 21 of the 31 Iranian provinces [5]. In this article, we review the published research on the parasites of camels in Iran from 1931 to February 2017.

## Methods

The authors checked all available documents on each of the search terms which included a combination of Iran or Iranian – in Persian and English – with one of the generic names of the camel parasites as mentioned in reference books “The Camel in Health and Diseases” [55], “Infectious Diseases in Camelids”, 2nd edition [171], and “Camelid Infectious Disorders” [172]. The databases and search engines employed for the present literature review were those of PubMed (www.pubmed.gov), Google (www.google.com), Scientific Information Database of Iran (www.sid.ir) and the collection of defended theses at all Iranian Universities (www.irandoc.ac.ir).

## Protozoal infections

### Trypanosomosis

*Trypanosoma evansi* is the most pathogenic and economically important protozoan parasite of camels that causes severe disease (surra) throughout camel rearing areas of the world [19]. *T. evansi* is now considered as an emerging zoonotic parasite [43]. *Trypanosoma* was first reported in Persia in 1876 and was known to be fatal for horses, according to Haig (reviewed by Lingard [72]). After massive mortality of more than 3000 horses in 1930 in the south of Iran, *Trypanosoma evansi*-like parasites were diagnosed in the blood of diseased animals [26]. *T. evansi* infection in camels in Iran was confirmed for the first time in 1935 by Delpy and Rafyi, and experimental infections of several mammals with *T. evansi* isolated from an infected camel were performed [30, 160]. The therapeutic and prophylactic efficacy of different dosages of Naganol^®^ (suramin sodium) and antrycide methyl sulphate was studied in naturally infected camels since the dose of Naganol^®^ recommended by the manufacturer was extremely expensive at that time [13, 123]. Since Iran does not lie within the tsetse belt, trypomastigotes in camels have usually been assigned to *T. evansi* according to their morphological and morphometric features upon microscopic examination. Prevalence rates between 0 and 19.47% for *Trypanosoma* infections have been reported in camels. However, there are only two sequence-confirmed studies on camel *T. evansi* infections from Iran (Table 1). In Iran, there is also one report of a natural *T. evansi* infection of a two-humped camel (*Camelus bactrianus*) [130]. Outbreaks of Trypanosomosis in dromedary herds associated with mortalities and abortions have been documented [31, 178]. Although no study has been conducted to examine infestation with known *T. evansi* vectors in the country, reports of natural infections with *T. evansi* in three dogs in Tehran [59], one horse in Shiraz [15], and one water buffalo in Ahvaz [135] with no history of travel, show that non-cyclic transmission occurs.

**Table 1.** Prevalence rates of *Trypanosoma evansi* infection in dromedary camels in Iran.

Number of tested animals	Prevalence (%)	Method	Reference and year*
127	9.5	LM	[14] 1979
196	7.7	LM	[124] 1995
37	5.4	LM	[85] 1995
333	9.5	LM	[180] 2000
60	1.6	LM	[120] 2006
285	14	LM	[88] 2009
262	0.4	LM	[24] 2009
113	19.5	LM	[129] 2009
110	15.5	LM	[145] 2011
117	3.4	LM + PCR + Seq	[117] 2013
21	4.8	LM	[6] 2014
278	1.1	PCR	[80] 2014
95	2.1	LM + PCR	[69] 2015
227	10.6	LM	[61] 2015
100	0.0	LM	[75] 2015
200	0.5	LM + PCR + Seq	[143] 2016
300	19	LM + PCR	[178] 2017
113	6.2	LM + PCR	[179] 2017

* Year of publication, LM: light microscopy, PCR: polymerase chain reaction, Seq: sequencing of PCR products.

### Eimeriosis

Coccidiosis of camels is an intestinal protozoan infection caused by apicomplexan parasites of the genus *Eimeria*. Disease caused by these parasites is of great economic importance because of losses due to enteritis, diarrhoea and poor weight gain [172]. Camels harbour their own species of *Eimeria* including *E. cameli*, *E. rajasthani*, *E. bactriani*, *E. dromedarii*, *E. pellerdyi* and *E. leuckarti* in OWC [71, 172]. Data on the frequency and diversity of *Eimeria* species in dromedaries and Bactrian camels are limited to seven studies which were based on coproscopy, except one that reported tissue alterations caused by *Eimeria* spp. [68]. Overall prevalences varying between 9.51% and 63% have been reported (Table 2). All reports concerned dromedary camels, except one that included Bactrian camels [174].

**Table 2.** Prevalence rates of *Eimeria* in camels in Iran.

Number of examined animals	Prevalence (%)	Camel species	Diversity of *Eimeria* species (%)	Reference and year*
100	63.0	Dromedary	NS	[68] 2000
125	12.8	Bactrian and Dromedary	*E. cameli* (11.1%), *E. bactriani* (42.2%), *E. rajasthani* † (26.7%), *E. pellerdyi* ‡ (15.6%), *E. dromedarii* (4.4%)	[174] 2007
306	18.62	Dromedary	NS	[23] 2009
164	20.7	Dromedary	*E. cameli* (19.3%), *E. bactriani* (52.42%), *E. pellerdyi* (15.68%), *E. dromedarii* (12.59%)	[173] 2010
100	29.0	Dromedary	*E. cameli* (100%)	[65] 2012
305	9.51	Dromedary	*E. cameli* (47.5%), *E. bactriani* (10%), *E. dromedarii* (42.5%)	[144] 2012
100	24.0	Dromedary	*E. cameli* (100%)	[119] 2013

* Year of publication, NS: not stated,

† only in dromedary camels,

‡ only in Bactrian camels.

### Cryptosporidiosis

Cryptosporidiosis is one of the major zoonotic infections associated with food-borne and water-borne outbreaks [25]. *Cryptosporidium* spp. has a faecal-oral transmission route. A broad range of hosts including humans, and domestic and wild animals can be affected by pathological changes induced by this gregarine parasite. Several polymerase chain reaction (PCR)-based studies on Bactrian camels, llamas and alpacas have detected *C. andersoni*, *C. muris*, *C. parvum* and *C. ubiquitum* (reviewed by Robertson et al. [134]). However, all studies on *Cryptosporidium* infections in dromedary camels were based on microscopic modified Ziehl-Neelsen stained faecal smear examinations. Overall prevalences based on faecal samples from dromedaries varied between 0.5% and 37.9% (Table 3). *Cryptosporidium* was detected in one out of 396 [102] and 12 out of 100 [146] examined abomasum mucosa samples. Available data on clinical disease in camels are limited. A total number of 170 Iranian camel faecal samples were examined, 10% of them showed *Cryptosporidium*-like organisms [175]. Camel calves less than one year of age showed the highest prevalence (20%). Infected calves showed wasting, diarrhoea and debility, while older oocyst shedders showed no symptoms. Rare data on zoonotic transmission are available. In Yazd Province, 24 of 100 people in long-term contact with camels were diagnosed with *Cryptosporidium* spp. [146]. Infection was significantly higher in winter (32%) than in summer (16%). Several factors could contribute to seasonality. Oocyst shedding in herbivores is mostly observed during the cold months (autumn and winter). It can therefore be assumed that, on the one hand, low temperature increases oocyst viability, and on the other, during the cold months, animals usually spend more time inside the barn and the oocysts are protected from direct sunlight [25].

**Table 3.** Prevalence rates of *Cryptosporidium* spp. in camels in Iran.

Number of examined animals	Prevalence (%)	Reference and year*
396	3.3	[102] 1995
306	1.9	[23] 2009
103	37.9	[131] 2009
65	16.9	[100] 2010
300	20.3	[146] 2012
170	10.0	[175] 2012
85	2.4	[122] 2013
184	0.5	[151] 2016

* Year of publication.

### Toxoplasmosis

The protozoan parasite *Toxoplasma gondii* is an important zoonotic pathogen worldwide. Viable parasites have been isolated from edible tissues of camels [46]. Several epizootiological studies have been conducted on the detection of anti-*Toxoplasma* antibodies in sera of camels from Iran. The first report was most likely from camels in Fars Province with an infection rate of 17% of 100 tested animals [103]. Antibodies against *Toxoplasma* were present in 4.16% of 120 tested camels from the north-eastern region of Iran using an immunofluorescence antibody test [137]. In another study, 14.57% seropositivity was reported from 254 tested camels from the centre of Iran, using the modified agglutination test based on direct agglutination of fixed parasites with sera pretreated with 2-mercaptoethanol [51]. Discrepancies in the results could be due to differences in the techniques, the initial serum dilution, age of the examined camels and different environmental conditions. There are two PCR-based publications on blood samples randomly collected from dromedaries. In the first study performed on 50 animals, *Toxoplasma* could not be detected [148]; however, in the other research work, the parasite was found in eight out of 122 samples (6.6%) [62]. In a comprehensive study on raw milk from various species carried out in 2013 by Safarpoor Dehkordi et al., 3.12% of 160 examined dromedary milk samples were positive in cell cultivation and cat bioassay. Capture ELISA and PCR results showed infection rates of 1.87% and 2.5%, respectively [138].

### Neosporosis

Neosporosis is primarily a disease of cattle and dogs. Abortions caused by *Neospora caninum* have been reported in alpaca and llama [150]. However, despite the numerous reports on the presence of anti-*N. caninum* antibodies in camel sera, clinical disease has not been documented in OWCs. Antibodies against *N. caninum* have been reported from camels in Mashhad (5.83%) [137] and Isfahan (3.22%) [58] using an indirect fluorescent antibody test (IFAT), and also in camels in Yazd (3.94%) using a *Neospora* agglutination test [51].

### Sarcocystiosis

There have been several cases of intestinal *Sarcocystis* infection in humans in Iran, but there is little information about the parasites’ molecular characteristics [4]. Until very recently, there was confusion concerning *Sarcocystis* species in dromedary camels. However, structural investigations of *S. cameli* and *S. ippeni* microcysts by light and transmission electron microscopy in 2015 by Dubey et al. led to a reconsideration of *Sarcocystis* taxonomy. *Sarcocystis camelicanis*, *S. camelocanis* and *S. miescheri* were considered invalid. Dogs are the most likely definitive hosts as excretion of *Sarcocystis* sporocysts in the faeces of dogs fed camel meat was reported in several studies [33]. Recently, the first macroscopic sarcocysts from a one-humped camel were confirmed by Dubey et al. [34]. However, there are no reports of macroscopic findings of sarcocystiosis from camels in Iran. Studies on the prevalence and geographic distribution pattern of microcystic infection in the most common sites of infection (i.e. oesophagus, heart, diaphragm, limb muscle and masseter muscle) by the use of the muscle compression/squash method, pepsin/trypsin digestion method and histopathological examination revealed infection rates between 51.5% and 83.6% in at least one of the examined tissues (Table 4). In two ultrastructural and molecular characterisation studies of *Sarcocystis* isolated from dromedaries in Iran, *S. cameli* was identified, and a 600-bp specific band was amplified after PCR amplification with specific primers [36, 97]. Nothing is known about *Sarcocystis* infection in Bactrian camels in Iran. However, in Mongolia and Kazakhstan, *Sarcocystis* infections have been reported in Bactrian camels but without species differentiation [172].

**Table 4.** Prevalence rates of *Sarcocystis* spp. in camels in Iran.

Number of examined animals	Overall	Oesophagus	Myocardium	Diaphragm	Tongue	Striated muscles	Reference and year*
39	52.6	46.1	52.6	24.0	n.i.	n.i.	[125] 1981
400	52.3	16.8	35.5	6.5	7.8	13.8	[159] 2006
250	83.6	58.8	48.0	46.8	28.0	41.6	[169] 2008
100	60.8	11.0	n.i.	17.0	15.0	n.i.	[57] 2010
130	51.5	55.22	50.8	n.i.	n.i.	38.8	[52] 2013

* Year of publication, n.i: not investigated.

### Besnoitiosis

Knowledge about besnoitiosis is scarce in camels and limited to only two documents reporting *Besnoitia* cysts in the intestine of dromedaries in India [64] and Iran [68]. In an article on camel diseases in Kenya, the authors stated that systematic besnoitiosis (which they referred to as globidiosis) occurred with clinical signs such as thickening of skin associated with hair loss, white patches on the cornea, nasal discharge, fever and acute (sometimes bloody) diarrhoea. In alimentary cases, acute (haemorrhagic) diarrhoea occurred, followed by rapid loss of condition, although no fever was reported. The fatality rate reached 10% [42]. In histopathological examination of the alimentary tract of 100 camels in Fars Province, Khodakaram Tafti et al. (2001) found *Besnoitia* parasites in 5% of the samples. In the mucosa of the jejunum and ileum of affected camels, a few small to large cysts with or without inflammatory reaction were seen [67].

### Theileriosis

The true role of *Theileria* parasites as tick-borne pathogens for camels is still not confirmed. So far, DNA of *Theileria equi*, *Theileria mutans*, *Theileria annulata* and *Theileria ovis* has been detected in peripheral blood of dromedaries worldwide [73, 118, 143, 168, 177]. However, it is still not clear whether these findings result from proliferation of *Theileria* in camels or transmission of blood parasites at the time of a tick bite. Clinical examination of naturally infected camels revealed fever, superficial lymph node swelling, loss of appetite, a sudden loss of condition and lacrimation. The morbidity rate was high with no lethal cases. Haematological and biochemical changes in sera of infected camels indicated that the disease greatly affects hepatic, renal and muscular functions [60]. In Iran, the most prominent hard tick species infesting camels is *Hyalomma dromedarii,* which is presumed to be a vector of *Theileria* spp. (see Tick infestation section). In this country, only two studies have reported intra-erythrocytic forms of the parasite in blood smears in 15.79% and 6.20% of the examined camels, respectively [54, 129]. The first report stated piroplasms in general, and did not differentiate between *Theileria* spp. and *Babesia* spp. However, in other studies, piroplasms and their developmental stages were not detected in peripheral blood or lymph nodes of the examined camels [24, 85, 180]. One report described successful treatment with buparvaquone in camels with *Theileria* piroplasms detected in blood smears and the typical signs of cattle *T. annulata* infection disappeared [53]. In three studies using PCR-based DNA detection and sequencing, *T. equi* and *T. annulata* were confirmed in the blood of randomly tested camels [16, 143], while in another study on the blood of 310 tick-infested camels by PCR, no positive animals were found [87].

### Babesiosis

Camels are not described as hosts of *Babesia* species and as with *Theileria* infection, no confirmed information is available about camel babesiosis due to the lack of experimental infections. However, typical signs of babesiosis such as fever, anaemia, haemoglobinuria, icterus and gastro-intestinal stasis have been documented in infected camels [162]. So far, the presence of DNA of *Babesia caballi* has been detected in camels [118], and this could have resulted from infection by infected ticks. There are only three reports on babesiosis in camels from Iran. In a light microscope study, the parasite was found in 3.54% of 113 examined blood samples [129]. In another report, *Babesia* DNA was found in eight out of 122 randomly tested camels in Iran, albeit without further analysis to species level [63]. Finally, based on PCR and sequencing methods, *B. caballi* was diagnosed in dromedaries of Iran [45]. Other investigators did not detect *Babesia* in the peripheral blood of tested animals, whether by light microscopy in 262, 37 and 333 samples, respectively [24, 85, 180] or by PCR [143] (*n* = 200).

### Balantidiosis

*Balantidium* spp. are often seen in the lumen of the caecum and large intestine of several mammals, such as swine, humans and non-human primates. This ciliated protozoan may cause a zoonotic disease [149]. Occurrence of an outbreak of human balantidiasis was reported in 1948 in the south of Iran by McCarey. Due to their religious beliefs, none of the 87 patients had contact with pigs. *Balantidium* could not be detected in intestinal contents of sheep, cattle or goats or in river water [78]. The investigator did not suspect the numerous camels as a possible source of zoonotic infection [28]. Pathologic examination of the intestinal lesions in 100 slaughtered camels showed that one of the most frequent findings was balantidiasis of the caecum and colon with a frequency of 19% [67]. In another study, *B. coli* trophozoites were observed in six out of 28 smears from the mucosa of the ileocaecal junction of camels [85]. Numerous *B. coli* trophozoites and cysts (15,000/g) without any other parasites were observed in the faeces of an anorexic dromedary with acute diarrhoea. A therapeutic regimen, which included intramuscular antibiotic therapy (ampicillin) and anti-inflammatory drugs (flunixin meglumine), was successful [167]. It is worth noting that under certain circumstances like being under stress, camels might shed ciliate trophozoites in faeces that can be confused with *Balantidium*. Further research is needed to confirm the pathological significance of these ciliates in camels.

## Helminthoses

### Helminthoses of the digestive system

The helminth fauna of the digestive tract of camelids is particularly rich with more than 50 species. Symptoms and signs of gastro-intestinal helminths in camels are numerous. The most typical ones are loss of weight, gastritis and/or enteritis, diarrhoea, anaemia and death [29]. Some of these helminths can cause zoonosis [79]. Infection rates of 78%–100% of the examined camels with at least one helminth species are reported from Iran. *Camelostrongylus mentulatus*, *Trichostrongylus probolurus*, *Haemonchus contortus*, *Haemonchus longistipes* and *Stilesia globipunctata* are the most common reported helminth species in different studies [10, 22, 37, 75, 84]. Reported helminths from slaughtered dromedaries and their isolation sites are listed in Table 5. There is some debate over the naming of *Nematodirella longissimespiculata* (syn. *Nematodirus longispiculata*, *Nematodirus alcidis*, *Nematodirus alcides*) in the literature but this name was kept as the most commonly used one.

**Table 5.** Helminth species recovered from the digestive system of dromedaries in Iran.

Species	Infection site	Reference
Trematoda		
Paramphistomatidae	Small intestine	[84]
*Fasciola hepatica* Linnaeus, 1758	Bile ducts	[35]
*Fasciola gigantica* Cobbold, 1855	Bile ducts	[81, 163]
Cestoda		
*Stilesia globipunctata* (Rivolta, 1874)	Small intestine	[10, 22, 37, 75, 84, 120]
*Moniezia benedeni* (Moniez, 1879)	Small intestine	[22, 37, 75, 84, 120]
*Moniezia expansa* (Rudolphi, 1810)	Small intestine	[10, 22, 75, 120]
*Echinococcus granulosus* sensu lato (Batsch, 1786)	Liver	(see Echinococcosis section)
Nematoda		
*Gongylonema pulchrum* Molin, 1857	Oesophagus	[84]
*Ascarops strongylina* (Rudolphi, 1819)	Abomasum	[84]
*Camelostrongylus mentulatus* (Railliet and Henry, 1909)	Abomasum	[10, 22, 37, 75, 84]
*Haemonchus contortus* (Rudolphi, 1803)	Abomasum	[10, 84, 120]
*Haemonchus longistipes* Railliet and Henry, 1909	Abomasum	[10, 22, 75, 84]
*Haemonchus tataricus* Evranova, 1940	Abomasum	[10, 37]
*Impalaia tuberculata* Mönnig, 1924	Abomasum	[10, 37]
*Marshallagia marshalli* (Ransom, 1907)	Abomasum	[10, 22, 37, 84]
*Parabronema skrjabini* Rassowska, 1924	Abomasum	[10, 22, 75, 84]
*Physocephalus sexalatus* (Molin, 1860)	Abomasum	[10, 37, 75, 84]
*Trichostrongylus probolurus* (Railliet, 1896)	Abomasum	[75]
*Teladorsagia circumcincta* (Stadelman, 1894)	Abomasum	[22]
*Teladorsagia circumcincta* (Stadelman, 1894)	Small intestine	[84]
*Nematodirella cameli* (Rajevskaja and Badanin, 1933)	Small intestine	[10, 22, 75, 84, 89]
*Nematodirella longissimespiculata* (Romanovich, 1915)	Small intestine	[10, 37, 75, 84, 89]
*Nematodirus oiratianus* Rajewskaja, 1929	Small intestine	[22, 75]
*Cooperia oncophora* (Railliet, 1898)	Small intestine	[22]
*Cooperia pectinata* Ransom, 1907	Small intestine	[10, 37]
*Nematodirella dromedarii* (May, 1920)	Small intestine	[22]
*Nematodirus abnormalis* May, 1920	Small intestine	[10, 37]
*Nematodirus dromedarii* May, 1920	Small intestine	[10, 37]
*Nematodirus helvetianus* May, 1920	Small intestine	[10, 37, 89]
*Nematodirus mauritanicus* Maupas and Seurat, 1912	Small intestine	[10]
*Nematodirus spathiger* (Railliet, 1896)	Small intestine	[10, 37, 89]
*Trichostrongylus axei* (Cobbold, 1879)	Small intestine	[37]
*Trichostrongylus colubriformis* (Giles, 1892)	Small intestine	[10, 22, 37]
*Trichostrongylus hamatus* Daubney, 1933	Small intestine	[10]
*Trichostrongylus probolurus* (Railliet, 1896)	Small intestine	[10, 22, 37]
*Trichostrongylus vitrinus* Looss, 1905	Small intestine	[10, 22, 37]
*Chabertia ovina* (Fabricius, 1794)	Large intestine	[84]
*Oesophagostomum radiatum* Rudolphi, 1803	Large intestine	[10, 37]
*Oesophagostomum venulosum* Rudolphi, 1809	Large intestine	[10, 84]
*Trichuris barbetonensis* Ortlepp, 1937	Large intestine	[22]
*Trichuris globulosa* (Linstow, 1901)	Large intestine	[10, 22, 37, 84]
*Trichuris infundibulus* (Linstow, 1906)	Large intestine	[10]
*Trichuris lani* (Artjuch, 1948)	Large intestine	[10, 37, 84]
*Trichuris skrjabini* Baskakov, 1924	Large intestine	[10, 84]
*Trichuris tenuis* Chandler,1930	Large intestine	[84]
*Trichuris vulpis* (Froelich, 1789)	Large intestine	[84]
*Trichuris raoi* Alwar and Achutan, 1960	Large intestine	[84]
*Trichuris cameli* (Rudolphi, 1819)	Large intestine	[84]

In the only study on the occurrence of gastro-intestinal helminths in Bactrian camels in Iran, Tajik et al. (2011) detected eggs of *Strongyloides* spp., *Marshallagia* spp., *Nematodirus* spp., *Trichuris* spp. and *Moniezia* spp. in the faeces of 50 sampled animals [166].

### Fasciolosis

Fasciolosis is a parasitic infection with global distribution, causing significant losses in domestic animal production, and it is an important food-borne trematode infection of increasing concern [107]. Both *Fasciola hepatica* and *F. gigantica* have been reported from several provinces of Iran and in different genera of snails [12]. In the sole specific study on the prevalence and pathology of *Fasciola* spp. in dromedaries of Iran, Eslami et al. (2003) examined the livers of 409 slaughtered camels and found that 5.3% of animals harboured *F. hepatica* flukes with an average number of 10.5 parasites per animal [35]. In other studies on liver infection of camels, only one out of 94 examined carcasses was infected with *Fasciola* [176] or no infected carcasses were found [94]. *F. gigantica* has also been isolated from camels in Iran [81, 163].

### Helminthoses of other organ systems

#### Echinococcosis

*Echinococcus* species are highly prevalent and human cystic echinococcosis is hyperendemic in Iran, with a human infection rate of 0.6–1.2/100,000 [136]. Since the study of Alavi and Maghami in 1963, numerous studies on the prevalence of echinococcosis/hydatidosis in different organs of slaughtered camels reported overall rates varying between 7.45% and 70%. Most of the studies examined the lungs and livers of camels; however, hydatid cysts were also found in the spleen and kidneys [3, 7, 8, 20, 21, 41, 56, 70, 75, 83–86, 90, 92, 94, 98, 147, 176]. The sequence analysis of metacestode isolates collected from camels indicated that *E. granulosus* sensu stricto (formerly G1 and G3 genotypes) and *E. canadensis* (formerly G6 genotype) infect dromedaries in Iran [93, 155–158, 161].

#### Lungworm infection

Several nematodes are able to infect the lower respiratory tract of domestic animals, usually resulting in bronchitis or pneumonia, or both [18]. Although *Dipetalonema evansi* might be observed in pulmonary arteries (see Dipetalonemosis section), two species of *Dictyocaulus filaria* and *Dictyocaulus viviparus* (syn. *Dictyocaulus cameli*) are found in the respiratory tract and lungs of dromedaries worldwide [172]. *Dictyocaulus filaria* and *D. viviparus* are not camel-specific and their occurrence in camels could have resulted from cross-infection from ruminants. However, as larvae need moist conditions for survival, lungworm infections are not considered a problem in hot and dry climates. In Iran, *D. filaria* was isolated from the lungs of 3.3% and 10% of examined dromedaries in two studies [84, 120].

#### Eyeworm infection

Helminths that affect the animals’ eyes may also cause human infections and are therefore major threats to human communities [112]. So far, *Thelazia leesei* and *T. rhodesi* have been reported in OWCs [171]. In the only report on eyeworm infections in camels in Iran, Vosooghi Afshar (1976) examined 400 eyes from slaughtered dromedaries in Tehran for the presence of *Thelazia* parasites. In total, 70 adult *T. leesei* worms were isolated. The number of worms varied between three and 10 per infected eye [170].

#### Dipetalonemosis

*Dipetalonema evansi* (syn. *Deraiophoronema evansi*) is the sole filarioid helminth believed to cause clinical disease in camels. The mature worm is typically observed in the testicles, epididymis, spermatic cord, lungs and heart. The sheathed microfilariae can be present in the peripheral bloodstream. Moderate infections are generally asymptomatic; however, severe infections might cause respiratory symptoms, emaciation, apathy, pale mucous membranes, orchitis, aneurysm of the spermatic cord, arteriosclerosis, heart malfunction and nervous impairments [110]. Adult worms have been isolated from the lungs and testicles of camels, and pathological findings described [84, 90, 98, 106, 110, 141]. Microfilariae of *D. evansi* were reported from blood samples in 0.88%–46.7% of the studied camels [24, 61, 85, 98, 110, 124, 129, 141]. Recently, *D. evansi* was detected in the blood of 8% of 200 examined camels by PCR and sequencing methods [142]. In this study, analysis of a cytochrome C oxidase subunit I (COI) sequence of filaroid nematodes showed paraphyly of *Dipetalonema evansi* and *Dipetalonema gracile*. Further investigations on different gene loci will clarify this nematode’s taxonomic position.

#### Onchocercosis

Some filarioid species represent major threats for human and animal health and cases of zoonotic onchocercosis are increasingly being reported worldwide [113]. In camels, adult *Onchocerca* parasites are commonly observed in connective tissues, while microfilariae are found in the dermis and occasionally circulating in peripheral blood. Reports on the prevalence and pathology of *Onchocerca fasciata* infection in dromedaries indicate that 5.82%–48% of the examined camels had skin lesions due to these filarial worms [11, 38, 40, 66, 75, 85, 91].

## Arthropod infections

### Tick infestation

Ectoparasites are not critical limiting factors for camel health; however, their presence can affect their productivity, which in turn has economic consequences by reducing animal weight gain and milk yield. Moreover, ticks transmit pathogens that affect animals and human populations. Ticks are abundant on camels of Iran and infestation rates of 9%–85.5% of examined dromedaries have been observed in different studies. Average numbers of 1.27–81.5 ticks per camel were recorded [27, 47, 95, 99, 101, 104, 128, 140]. Reported tick species from dromedaries are enlisted in Table 6.

**Table 6.** Tick species collected from dromedaries according to their overall frequency in Iran.

Tick species	Rank	Reference
*Hyalomma dromedarii* Koch, 1844	1	[1, 2, 27, 44, 47, 75, 77, 95, 99, 101, 104, 114, 128, 139, 140]
*Hyalomma anatolicum* Koch, 1844	2	[2, 27, 44, 47, 75, 77, 95, 99, 101, 104, 128, 139]
*Hyalomma schulzei* Olenev, 1931	3	[2, 44, 47, 75, 77, 95, 114, 128]
*Hyalomma marginatum* Koch, 1844	4	[27, 44, 77, 101, 104, 114, 139]
*Hyalomma asiaticum* Schulze and Schlottke, 1930	5	[27, 47, 95, 101, 139]
*Hyalomma scupense* Schulze, 1919	5	[2, 75, 95, 128]
*Hyalomma impeltatum* Schulze and Schlottke, 1930	6	[2, 77, 95, 101]
*Rhipicephalus turanicus* (Pomerantsev et al., 1940)	7	[47, 75, 95]
*Hyalomma excavatum* Koch, 1844	7	[1, 47, 95]
*Rhipicephalus bursa* (Canestrini and Fanzago, 1878)	8	[101, 128]
*Rhipicephalus sanguineus* (Latreille, 1806)	8	[47, 77]
*Hyalomma lusitanicum* Koch, 1844	9	[1]
*Argas lahorensis* Neumann, 1908	9	[128]

The rank of the tick species goes from 1 (the most frequent) to 9 (least frequent).

### Mange mite infection

Camels are affected by a range of mites including *Sarcoptes scabiei*, *Psoroptes* spp., *Chorioptes* spp. and *Demodex* spp. [172]. Mange was a major nuisance for the camels of caravans in past times. Camel handlers used to apply tar, turpentine or “wild rocket” oil on the body of the animals. These treatments were repeated until the problem was resolved [164]. Sarcoptic mange is regarded as one of the most prevalent diseases of camels and can also be transmitted to humans. Although mange is common in camels of Iran (A. Sazmand, personal observation) there are few published reports on the disease, and usually without specification of the causative mite species [9, 85]. However, *Sarcoptes scabiei* var. *cameli* has been identified in some cases [76]. During examination of the eyelid of domestic herbivores in Iran, Rak and Rahgozar (1975) found demodectic mange infection in 23 out of 153 investigated dromedaries (15%), with no significant histological changes other than distension of the hair follicle [127].

### Biting and nuisance flies

Although fly-borne parasitic diseases such as trypanosomosis and nasal bot infection are common, there is a paucity of information about the flies affecting camels. Various species of the Tabanidae and Muscidae families are observed according to a previous report [74] and also the first author’s personal observations.

### Myiasis

Nasopharyngeal myiasis caused by Oestridae is very common in old world camelids. The camel nasal bot, *Cephalopina titillator*, is usually found at necropsy or during meat inspection, and infection rates of up to 80.72% of the examined animals have been reported from Iran [74, 75, 85, 109, 111, 120, 126, 132, 152]. *C*. *titillator* was also found in the lungs of four out of 40 examined dromedaries in Iran [109]. There are also two reports of genital and gingival myiasis caused by *Wohlfahrtia magnifica*. Genital myiasis was recorded around the perineum and vagina of five out of 35 camels in a herd in the southwest of Iran [115]. A single case of gingival myiasis was reported in a 15-year-old camel during inspection of the teeth of slaughtered camels in Mashhad [96].

### Linguatulosis

*Linguatula serrata*, the nose worm of canids, is a well-known zoonotic parasite [32]. The larval stage of *L. serrata* has been diagnosed in mesenteric and mediastinal lymph nodes, and in the livers and lungs of dromedaries. Up to 162 nymphs were collected by Majidi Rad et al. (2015) from one infected camel [75] (Table 7). There is also one report of *L. serrata* nymphs in the lungs of a two-humped camel in Iran [50]. One recent paper investigated phylogenetic relationships among seven *L. serrata* isolates collected from camels, cattle, goats, sheep and dogs of Iran. Neither host species nor geographical location was associated with genotypes [48].

**Table 7.** Prevalence in percent of linguatuliasis in camels in Iran.

Number of examined animals	Mesenteric and mediastinal lymph nodes	Liver	Lungs	Reference and year*
40	12.5	n.i.	n.i.	[109] 1993
100	5.0	n.i.	n.i.	[67] 2001
103	75.0	30.4	29.7	[165] 2007
200	35.0	11.5	n.i.	[116] 2007
400	21.0	4.5	n.i.	[153] 2008
140	13.5	1.4	1.4	[49] 2010
210	16.2	n.i.	n.i.	[121] 2010
101	12.9	n.i.	n.i.	[108] 2011
400	18.25	n.i.	n.i.	[105] 2012
232	21.12	n.i.	n.i.	[133] 2012
185	13.5	n.i.	n.i.	[82] 2013
132	20.5	n.i.	n.i.	[17] 2014
213	64.7	n.i.	n.i.	[75] 2015
272	15.1	1.8	n.i.	[39] 2016

* Year of publication, n.i: not investigated.

## Conclusion

Camels play an important role in the epidemiology of parasitic diseases under the three aspects of animal health, transmission to other livestock and zoonoses. Parasitic infections of camels may cause reduced milk and meat production, impaired fertility and decreased calving rates. They may also lower the working efficiency or even result in death and consequently high economic losses (e.g. in camels suffering from surra). As a result, there is a need for an integrated control programme against economically important parasites of camels as well as to include parasites in health surveillance of camels. Several species of the order *Strongylida* can infect both camels and ruminants, and ticks with a low host specificity can be shared by several hosts (e.g. *Hyalomma dromedarii*) and transmit diseases between them. Consequently, deworming and tick control programmes for camels (as well as for affected livestock) are recommended to avoid cross-infections in mixed farming. Concerning the public health importance of camel parasites, several protozoa (e.g. *T. evansi*) and helminths (e.g. *F. hepatica*) may be transmitted to humans through close contact with infected camels or indirectly via invertebrate vectors in their surroundings, or via consumption of infected organs of camels. Therefore, surveillance of camel health as well as improving the community’s knowledge of public health issues in this regard are necessary.

The present work reflects the current state of knowledge on the parasitic fauna of camels in Iran. This knowledge is, however, probably not exhaustive because it was based on clinical and scientific reports and the experience of the authors. Other camel parasites may be present in Iran since they may not have been detected so far and included in published reports, for various reasons. This review will also serve as a reference for future research activities. Detailed epidemiological studies on the parasites of camels strongly call for molecular diagnostic tools for proper classification of species and genotypes to improve the existing diagnostic tools and give more detailed insight into the epidemiology, transmission and risk factors of camel parasites.

## Conflict of interest

The authors declare that there is no conflict of interest.

## References

[1] Abbassian-Lintzen R. 1960 A preliminary list of ticks (Acarina: Ixodidae) occurring in Iran and their distributional data. Acarologia, 2, 43–61.

[2] Abbassian-Lintzen R. 1961 Records of ticks (Acarina: Ixodidae) from southest Iran (Iranian Baluchistan and the Jiroft area). Acarologia, 3, 546–559. 13680911

[3] Afshar A, Nazarian I, Baghban-Baseer B. 1971 A survey of the incidence of hydatid cyst in camels in south Iran. British Veterinary Journal, 127(11), 544–546. 5167826 10.1016/s0007-1935(17)37288-3

[4] Agholi M, Taghadosi Z, Mehrabani D, Zahabiun F, Sharafi Z, Motazedian MH, Hatam GR, Naderi Shahabadi S. 2016 Human intestinal sarcocystosis in Iran: there but not seen. Parasitology Research, 115(12), 4527–4533. 27637226 10.1007/s00436-016-5244-6

[5] Agriculture-Jahad Ministry of Iran. 2016 Annual production report. Tehran, Iran: Department of Animal Production, Agriculture-Jahad Ministry of Iran [in Persian].

[6] Ahmadi Hamedani M, Ghazvinian K, Darvishi MM. 2014 Hematological and serum biochemical aspects associated with a camel (*Camelus dromedarius*) naturally infected by *Trypanosoma evansi* with severe parasitemia in Semnan, Iran. Asian Pacific Journal of Tropical Biomedicine, 4(9), 743–745.

[7] Ahmadi NA. 2005 Hydatidosis in camels (*Camelus dromedarius*) and their potential role in the epidemiology of *Echinococcus granulosus* in Iran. Journal of Helminthology, 79(2), 119–125. 15946390 10.1079/joh2005279

[8] Alavi A, Maghami G. 1963 L’échinococcose hydatidose en Iran. Revue Médicale du Moyen Orient, 3, 205–210. 14011635

[9] Amri A-A. 1940 Mite. DVM Dissertation, University of Tehran, [in Persian].

[10] Anvari Tafti MH, Sazmand A, Hekmatimoghaddam S, Moobedi I. 2013 Gastrointestinal helminths of camels (*Camelus dromedarius*) in center of Iran. Tropical Biomedicine, 30(1), 56–61. 23665708

[11] Anvari Tafti MH, Sazmand A, Hekmatimoghaddam S, Moobedi I. 2015 Prevalence and pathology of *Onchocerca* infection in camels (*Camelus dromedarius*) in central parts of Iran. Iranian Journal of Veterinary Medicine, 9(4), 257–261.

[12] Ashrafi K. 2015 The status of human and animal fascioliasis in Iran: a narrative review article. Iranian Journal of Parasitology, 10(3), 306–328. 26622287 PMC4662732

[13] Azmi M. 1938 Chemotherapy in trypanosomosis – study on efficacy of Naganol in camel trypanosomosis. MSc Dissertation, University of Tehran, [in Persian].

[14] Badamchi H. 1979 Haemoparasites of camels in slaughterhouse of Tehran. DVM Dissertation, University of Tehran, [in Persian].

[15] Badiei K, Ahmadi MR, Nazifi S. 1998 (Equine trypanosomiasis (*T. evansi*): a case report associated with abortion). Iranian Veterinary Journal, 1(2), 69–75, [in Persian].

[16] Bahrami S, Tabandeh MR, Nikbin A, Alborzi AR, Ghadrdan AR. 2016 Prevalence and phylogenetic analysis of *Theileria equi* in Iranian dromedaries. Archives of Razi Institute, 71(3), 169–175.

[17] Bamorovat M, Zarandi MB, Mostafavi M, Kheirandish R, Sharifi I, Radfar MH. 2014 The prevalence of *Linguatula serrata* nymphs in mesenteric and mediastinal lymph nodes in one-humped camels (*Camelus dromedarius*) slaughtered in Rafsanjan slaughterhouse, Iran. Journal of Parasitic Diseases, 38(4), 374–377. 25320486 10.1007/s12639-013-0258-9 PMC4185030

[18] Beugnet F, Bourdeau P, Chalvet-Monfray K, Cozma V, Farkas R, Guillot J, Halos L, Joachim A, Losson B, Miró G, Otranto D, Renaud M, Rinaldi L. 2014 Parasites of domestic owned cats in Europe: co-infestations and risk factors. Parasites & Vectors, 7, 291. 24965063 10.1186/1756-3305-7-291 PMC4082530

[19] Boid R, Jones TW, Luckins AG. 1985 Protozoal diseases of camels. British Veterinary Journal, 141(1), 87–105. 4039614 10.1016/0007-1935(85)90132-0

[20] Borji H, Azizzadeh M, Afsai A. 2011 An abattoir-based study of hydatidosis in the dromedary (*Camelus dromedarius*) in Mashhad, Iran. Journal of Helminthology, 85(4), 478–479. 21251343 10.1017/S0022149X10000933

[21] Borji H, Parandeh S. 2010 The abattoir condemnation of meat because of parasitic infection, and its economic importance: results of a retrospective study in north-eastern Iran. Annals of Tropical Medicine and Parasitology, 104(8), 641–647. 21144183 10.1179/136485910X12851868780261

[22] Borji H, Razmi GR, Movassaghi AR, Maleki M. 2010 A study on gastrointestinal helminths of camels in Mashhad abattoir, Iran. Iranian Journal of Veterinary Research, 11(2), 174–179.

[23] Borji H, Razmi GR, Movassaghi AR, Naghibi A, Maleki M. 2009 Prevalence of *Cryptosporidium* and *Eimeria* infections in dromedary (*Camelus dromedarius*) in abattoir of Mashhad, Iran. Journal of Camel Practice and Research, 16(2), 167–170.

[24] Borji H, Razmi GR, Parandeh S. 2009 Epidemiological study on haemoparasites of dromedary (*Camelus dromedarius*) in Iran. Journal of Camel Practice and Research, 16(2), 217–219.

[25] Cacciò SM, Widmer G. 2013 *Cryptosporidium*: parasite and disease. Vienna: Springer-Verlag.

[26] Carpentier G-A-L-E. 1931 Les services vétérinaires en Perse. Paris: Librairie Le François.

[27] Champour M, Chinikar S, Mohammadi G, Razmi G, Shah-Hosseini N, Khakifirouz S, Mostafavi E, Jalali T. 2016 Molecular epidemiology of Crimean-Congo hemorrhagic fever virus detected from ticks of one humped camels (*Camelus dromedarius*) population in northeastern Iran. Journal of Parasitic Diseases, 40(1), 110–115. 27065608 10.1007/s12639-014-0458-y PMC4815851

[28] Cox FEG. 2005 Human balantidiasis in Iran: are camels reservoir hosts? Trends in Parasitology, 21(12), 553, author reply 554–555. 16202655 10.1016/j.pt.2005.09.002

[29] Dakkak A, Ouhelli H. 1987 Helminths and helminthoses of the dromedary. A review of the literature. Revue Scientifique et Technique (International Office of Epizootics), 4(1), 447–461. 10.20506/rst.6.2.302 32370332

[30] Delpy L, Rafyi A. 1947 La trypanosomiase du dromadaire en Iran, étude experimentale de *Trypanosoma evansi* (Steel, 1885). Archives of Razi Institute, 5(1), 33–50.

[31] Derakhshanfar A, Mozaffari AA, Zadeh AM. 2010 An outbreak of trypanosomiasis (Surra) in camels in the Southern Fars province of Iran: clinical, hematological and pathological findings. Research Journal of Parasitology, 5(1), 23–26.

[32] Dhaliwal BBS, Juyal PD. 2013 Parasitic Zoonoses. New Delhi: Springer India.

[33] Dubey JP, Hilali M, Van Wilpe E, Calero-Bernal R, Verma SK, Abbas IE. 2015 A review of sarcocystosis in camels and redescription of *Sarcocystis cameli* and *Sarcocystis ippeni* sarcocysts from the one-humped camel (*Camelus dromedarius*). Parasitology, 142(12), 1481–1492. 26416197 10.1017/S0031182015000852

[34] Dubey JP, A’aji NN, Mowery JD, Verma SK, Calero-Bernal R. 2017 Identification of macroscopic sarcocysts of *Sarcocystis cameli* from one-humped camel (*Camelus dromedarius*) in Iraq. Journal of Parasitology, 103(2), 168–169. 28121491 10.1645/16-135

[35] Eslami A, Ranjbar Bahadori S, Eskandari A, Sedaghat R. 2003 Study on the prevalence and pathology of *Fasciola* in camels (*Camelus dromedarius*) of Iran. Journal of Faculty of Veterinary Medicine University of Tehran, 58(2), 97–100, [in Persian with English abstract].

[36] Eslampanah M, Motamedi GR, Dalimi A, Noori A, Habibi GR, Aghaeepour K, Niroumand M. 2016 Study of camel and goat *Sarcocystis* by electron microscopic and PCR-RFLP. Pajouhesh & Sazandegi, 29(3), 77–84, [in Persian with English abstract].

[37] Etminan S. 1995 Study of intestinal helminths in slaughtered camels in Yazd slaughterhouse. MSc Dissertation, Tehran University of Medical Sciences, [in Persian].

[38] Farjanikish G, Namazi F, Rajabloo M. in press Prevalence and pathological lesions of onchocercosis (*Onchocerca fasciata*) in camels (*Camelus dromedarius*). Bulgarian Journal of Veterinary Medicine, DOI: 10.15547/bjvm.1013

[39] Farjanikish G, Shokrani H. 2016 Prevalence and morphopathological characteristics of linguatulosis in one-humped camel (*Camelus dromedarius*) in Yazd, Iran. Parasitology Research, 115(8), 3163–3167. 27106238 10.1007/s00436-016-5074-6

[40] Farkhondeh S. 1980 Dirofilariosis in camels of Iran. DVM Dissertation, University of Tehran, [in Persian].

[41] Fathi S, Mirzaei Dehaghi M, Radfar MH. 2011 Occurrence of hydatidosis in camels (*Camelus dromedarius*) and their potential role in the epidemiology of *Echinococcus granulosus* in Kerman area, southeast of Iran. Comparative Clinical Pathology, 21(5), 921–927.

[42] Fazil M, Hofmann R. 1981 Haltung und Krankheiten des Kamels. Tieraerztliche Praxis, 9, 389–402. 7348946

[43] Fong IW. 2017 New and emerging parasitic zoonoses, in Emerging Zoonoses. Fong IW, Editor Cham, Switzerland: Springer International Publishing p. 211–239.

[44] Ganjali M, Dabirzadeh M, Sargolzaie M. 2014 Species diversity and distribution of ticks (Acari: Ixodidae) in Zabol County, eastern Iran. Journal of Arthropod-Borne Diseases, 8(2), 219–223. 26114136 PMC4478434

[45] Ganjali Tafreshi A. 2016 Phylogenetic analysis of camel piroplasmids in Iran based on 18s rRNA gene. MSc Dissertation, Shahid Chamran University of Ahvaz, [in Persian].

[46] Gebremedhin EZ, Yunus HA, Tesfamaryam G, Tessema TS, Dawo F, Terefe G, Di Marco V, Vitale M. 2014 First report of *Toxoplasma gondii* in camels (*Camelus dromedarius*) in Ethiopia: bioassay and seroepidemiological investigation. BMC Veterinary Research, 10, 222. 25266944 10.1186/s12917-014-0222-7 PMC4189602

[47] Ghashghaei O, Nourollahi Fard SR, Khalili M, Sharifi H. 2016 Abundance and associated risk factors of ixodid ticks (Acari: Ixodidae) collected from one-humped camels (*Camelus dromedarius*) in Sistan and Balouchestan region, southeast of Iran. Persian Journal of Acarology, 5(3), 219–227.

[48] Ghorashi SA, Tavassoli M, Peters A, Shamsi S, Hajipour N. 2016 Phylogenetic relationships among *Linguatula serrata* isolates from Iran based on 18S rRNA and mitochondrial cox1 gene sequences. Acta Parasitologica, 61(1), 195–200. 27149706

[49] Haddadzadeh H, Athari S, Abedini R, Khazraii Nia S, Khazraii Nia P, Nabian S, Haji-Mohamadi B. 2010 One-humped camel (*Camelus dromedarius*) infestation with *Linguatula serrata* in Tabriz, Iran. Iranian Journal of Arthropod-Borne Diseases, 4(1), 54–59. 22808389 PMC3385538

[50] Haddadzadeh H, Athari SS, Hajimohammadi B. 2009 The first record of *Linguatula serrata* infection of two-humped camel (*Camelus bactrinus*) in Iran. Iranian Journal of Parasitology, 4(1), 59–61.

[51] Hamidinejat H, Ghorbanpour M, Rasooli A, Nouri M, Hekmatimoghaddam S, Mohammad Namavari M, Pourmendi-Borojeni M, Sazmand A. 2013 Occurrence of anti-*Toxoplasma gondii* and *Neospora caninum* antibodies in camels (*Camelus dromedarius*) in the center of Iran. Turkish Journal of Veterinary and Animal Sciences, 37, 277–281.

[52] Hamidinejat H, Hekmatimoghaddam S, Jafari H, Sazmand A, Haddad Molayan P, Derakhshan L, Mirabdollahi S. 2013 Prevalence and distribution patterns of *Sarcocystis* in camels (*Camelus dromedarius*) in Yazd Province, Iran. Journal of Parasitic Diseases, 37(2), 163–165. 24431561 10.1007/s12639-012-0150-z PMC3793098

[53] Hamidinejat H, Razi Jalali MH, Nouri M. 2008 Report of clinical theileriosis in one-humped camel (*Camelus dromedarius*) in Khuzestan Province, Iran, in 15th Iran Veterinary Congress Tehran, Iran, [in Persian].

[54] Hekmatimoghaddam S, Sazmand A, Rasooli A, Hamidinejat H, Jafari H. 2012 Laboratory tests in dromedary camels naturally infected with piroplasms in Iran: study and review of literature. Journal of Camel Practice and Research, 19(2), 217–221.

[55] Higgins A. 1986 The camel in health and disease. London: Baillière Tindall.

[56] Hosseini SH, Bokaei S, Motevaselolhoseini M. 1999 Hydatid cyst and its role in epidemiology of *Echinococcus granulosus* in camel. Journal of Faculty of Veterinary Medicine University of Tehran, 53(3,4), 83–86, [in Persian with English abstract].

[57] Hosseini SR, Atayi A, Rahimi E, Jafarian M. 2010 Prevalence of sarcocystosis in slaughtered camels of Najaf Abad slaughterhouse. Journal of Veterinary Pathobiology, 1(2), 41–48, [in Persian with English abstract].

[58] Hosseininejad M, Pirali Kheirabadi K, Hosseini F. 2009 Seroprevalence of *Neospora caninum* infection in camels (*Camelus dromedarius*) in Isfahan Province, center of Iran. Iranian Journal of Parasitology, 4(4), 61–64.

[59] Hosseininejad M, Shirani D, Nabian S, Nassiri SM, Mazaheri R. 2006 *Trypanosoma evansi* in three dogs in Iran. Comparative Clinical Pathology, 16(1), 69–71.

[60] Ismael AB, Swelum AA, Khalaf AF, Abouheif MA. 2014 Clinical, haematological and biochemical alterations associated with an outbreak of theileriosis in dromedaries (*Camelus dromedarius*) in Saudi Arabia. Pakistan Veterinary Journal, 34(2), 209–213.

[61] Karimi A, Rahbari S, Yousefi A. 2015 Blood parasites of camels from central regions of Iran: comparative evaluation of various detection techniques and serum protein components. Journal of Advances in Parasitology, 2(1), 1–4.

[62] Khamesipour F, Doosti A, Iranpour Mobarakeh H, Komba EVG. 2014 *Toxoplasma gondii* in cattle, camels and sheep in Isfahan and Chaharmahal va Bakhtiary Provinces, Iran. Jundishapur Journal of Microbiology, 7(6), e17460. 25371809 10.5812/jjm.17460 PMC4217666

[63] Khamesipour F, Doosti A, Koohi A, Chehelgerdi M, Mokhtari-Farsani A, Chengula AA. 2015 Determination of the presence of *Babesia* species in blood samples of cattle, camel and sheep in Iran by PCR. Archives of Biological Sciences, 67(1), 83–90.

[64] Kharole MU, Gupta SK, Singh J. 1981 Note on besnoitiosis in a camel. Indian Journal of Animal Sciences, 51, 802–804.

[65] Kheirandish R, Nourollahi Fard SR, Faryabi Z. 2012 Prevalence and pathologic study of *Eimeria cameli* in slaughtered camels. Eurasian Journal of Veterinary Sciences, 28(3), 138–141.

[66] Khodakaram-Tafti A, Khordadmehr M. 2010 Gross and histopathological lesions of onchocercosis in camels (*Camelus dromedarius*). Journal of Camel Practice and Research, 17(1), 37–39.

[67] Khodakaram-Tafti A, Maleki M, Oryan A. 2001 Pathological study of intestines and mesentric lymph nodes of camels (*Camelus dromedarius*) slaughtered in Iran. Journal of Camel Practice and Research, 8(2), 209–213.

[68] Khodakaram-Tafti A, Maleki M, Oryan A, Mozaffari AA. 2000 Pathological study of digestive system lesions of camels (*Camelus dromedarius*) slaughtered in Iran. in Proceedings of 18th Meeting of the European Society of Veterinary Pathology, Amsterdam, The Netherlands.

[69] Khosravi A, Hakimi Parizi M, Bamorovat M, Borhani Zarandi M, Mohammadi MA. 2015 Prevalence of *Trypanosoma evansi* in camels using molecular and parasitological methods in the southeast of Iran, 2011. Journal of Parasitic Diseases, 39(3), 422–425. 26345045 10.1007/s12639-013-0355-9 PMC4554580

[70] Kojouri GA, Nourani H, Sadeghian S, Imani H, Raisi A. 2014 Pathological findings of slaughtered camels’ (*Camelus dromedaris*) kidneys in Najaf-Abad, Iran. Veterinary Research Forum, 5(3), 231–235. 25568724 PMC4279641

[71] Kumar S, Ghorui S, Patil NV. 2016 *Eimeria leuckarti* from dromedaries camel calves. Journal of Camel Practice and Research, 23(1), 91–94.

[72] Lingard A. 1893 Report on horse Surra. Bombay: Government Central Press.

[73] Lorusso V, Wijnveld M, Latrofa MS, Fajinmi A, Majekodunmi AO, Dogo AG, Igweh AC, Otranto D, Jongejan F, Welburn SC, Picozzi K. 2016 Canine and ovine tick-borne pathogens in camels, Nigeria. Veterinary Parasitology, 228, 90–92. 27692338 10.1016/j.vetpar.2016.08.014 PMC5061551

[74] Maghami G. 1968 External parasite of livestock in Iran. Archives of Razi Institute, 20, 81–83.

[75] Majidi Rad M, Hosseini SH, Rajabloo M, Nabian S, Gerami Sadeghian A. 2015 Parasites of one-humped camel (*Camelus dromedarius*) in Iran: an abattoir study. Journal of Camel Practice and Research, 22(2), 261–264.

[76] Makhsoos S. 1989 Study of camels’ mange (*Sarcoptes scabiei* var. *cameli*) in Abyek slaughterhouse of Qazvin. MSc Dissertation, Tehran University of Medical Sciences, [in Persian].

[77] Mazlum Z. 1971 Ticks of domestic animals in Iran: geographic distribution, host relation, and seasonal activity. Journal of Faculty of Veterinary Medicine University of Tehran, 27(1), 1–32, [in Persian with English abstract].

[78] McCarey AG. 1952 Balantidiasis in South Persia. British Medical Journal, 1(4759), 629–631. 14905008 10.1136/bmj.1.4759.629 PMC2023172

[79] McCarthy J, Moore TA. 2000 Emerging helminth zoonoses. International Journal for Parasitology, 30(12–13), 1351–1359. 11113260 10.1016/s0020-7519(00)00122-3

[80] Mehrabiyan S, Mahzounieh M, Rabbani-Khorasgani MTH, Amiri-Dehcheshmeh JA, Ghorbani A, Esmaili-Najafabadi H, Salimi M. 2014 Molecular detection of *Trypanosoma* from one-humped camels slaughtered in Najafabad slaughterhouse. Biological Journal of Microorganism, 3(10), 45–50, [in Persian with English abstract].

[81] Meshgi B, Eslami A, Hosseini SH, Hemmatzadeh F, Hooshmand E. 2007 Comparative assessment of electrophoretic patterns of *Fasciola* somatic antigens in different hosts. Pajouhesh & Sazandegi, 75, 156–159, [in Persian with English abstract].

[82] Mirzaei M, Rezaei H, Ashrafihelan J, Nematollahi A. 2013 The prevalence of *Linguatula serrata* nymphs in one-humped camel (*Camelus dromedarius*) in Northwest of Iran. Scientia Parasitologica, 14(1), 47–50.

[83] Mirzaei M, Rezaei H, Nematollahi A, Ashrafihelan J. 2016 Survey of hydatidosis infection in slaughtered camel (*Camelus dromedarius*) in Tabriz area, Northwest Iran. Journal of Parasitic Diseases, 40(2), 444–447. 27413318 10.1007/s12639-014-0523-6 PMC4927505

[84] Mirzayans A, Halim R. 1980 Parasitic infection of *Camelus dromedarius* from Iran. Bulletin de la Société de Pathologie Exotique et de ses Filiales, 73(4), 442–445. 7460130

[85] Mizan Zadeh H. 1995 Study of diversity and prevalence of occurance of diseases in slaughtered camels in Najaf-Abad slaughterhouse. DVM Dissertation, University of Tehran, [in Persian].

[86] Mobedi I, Madadi H, Arfaa F. 1970 Camel, *Camelus dromedarius*, as intermediate host of *Echinococcus granulosus* in Iran. Journal of Parasitology, 56(6), 1255. 5534033

[87] Moezi V, Sarani A, Hashemi H, Rasekh M. 2016 Molecular study of *Theileria camelensis* and *Theileria dromedarii* strains based on sequence of 18S ribosomal DNA fragment in camels. Journal of Fundamental and Applied Sciences, 8(2), 399–406.

[88] Moghaddar N, Dianatpour V. 2009 Distribution pattern of *Trypanosoma evansi* in camels (*Camelus dromedarius*) in Iran. Journal of Camel Practice and Research, 16(1), 73–75.

[89] Moghaddar N, Oryan A, Gorjipour S, Akbari M. 2010 Studies on seasonal prevalence and clinico-pathology of gastro-intestinal helminths of camel (*Camelus dromedarius*) in Iran with special reference to Nematodirinae nematodes. Journal of Camel Practice and Research, 17(2), 147–150.

[90] Moghaddar N, Oryan A, Hanif Pour MR. 1992 Helminths recovered from the liver and lungs of camel with special reference to their incidence and pathogenesis in Shiraz, Islamic Republic of Iran. Indian Journal of Animal Sciences, 62(11), 1018–1023.

[91] Moghaddar N, Zahedie A. 2006 Prevalence and pathogenesis of *Onchocerca fasciata* infection in camels (*Camelus dromedarius*) in Iran. Journal of Camel Practice and Research, 13(1), 31–35.

[92] Moghaddas E, Borji H, Naghibi A, Razmi G, Shayan P. 2014 Epidemiological study of hydatidosis in the dromedaries (*Camelus dromedarius*) of different regions of Iran. Asian Pacific Journal of Tropical Biomedicine, 4(Suppl 1), S148–S151. 25183070 10.12980/APJTB.4.2014C725 PMC4025301

[93] Moghaddas E, Borji H, Naghibi A, Shayan P, Razmi GR. 2015 Molecular genotyping of *Echinococcus granulosus* from dromedaries (*Camelus dromedarius*) in eastern Iran. Journal of Helminthology, 89(1), 100–104. 24018210 10.1017/S0022149X13000631

[94] Mohamadzadeh T, Shams T, Khanaliha S, Marhamatizadeh MH, Vafa A. 2016 A study on prevalence of some helminthic infections of the liver and lungs among ruminants in abattoir of Fars province, Iran. Archives of Razi Institute, 71(4), 245–251.

[95] Moshaverinia A, Moghaddas E. 2015 Prevalence of tick infestation in dromedary camels (*Camelus dromedarius*) brought for slaughter in Mashhad abattoir, Iran. Journal of Parasitic Diseases, 39(3), 452–455. 26345051 10.1007/s12639-013-0367-5 PMC4554562

[96] Moshaverinia A, Moghaddas E, Maleki M, Borji H. 2013 Gingival myiasis of camel (*Camelus dromedarius*) caused by *Wohlfahrtia magnifica*. Scientia Parasitologica, 14(2), 85–87.

[97] Motamedi GR, Dalimi A, Nouri A, Aghaeipour K. 2011 Ultrastructural and molecular characterization of *Sarcocystis* isolated from camel (*Camelus dromedarius*) in Iran. Parasitology Research, 108(4), 949–954. 21061021 10.1007/s00436-010-2137-y

[98] Mowlavi G, Massoud J, Mobedi I. 1997 Hydatidosis and testicular filariasis (*D. evansi*) in camel (*C. dromedarius*) in central part of Iran. Iranian Journal of Public Health, 26(1–2), 21–28.

[99] Najarnezhad V, Mishmast Z, Razmi G, Azad M. 2009 Identification of Ixodidae ticks of imported camels in Zabol area. Pajouhesh & Sazandegi, 85, 35–39, [in Persian with English abstract].

[100] Nazifi S, Behzadi MA, Haddadi SH, Raayat Jahromi A, Mehrshad S, Tamadon A. 2010 Prevalence of *Cryptosporidium* isolated from dromedary camels (*Camelus dromedarius*) in Qeshm Island, Southern Iran. Comparative Clinical Pathology, 19(3), 311–314.

[101] Nazifi S, Tamadon A, Behzadi M-A, Haddadi S, Raayat-Jahromi A-R. 2011 One-humped camels (*Camelus dromedaries*) hard ticks infestation in Qeshm Island, Iran. Veterinary Research Forum, 2(2), 135–138.

[102] Nouri M, Razmyar J, Keyhani P. 1995 A *Cryptosporidium muris* like parasite in large ruminants in various parts of Iran. Journal of Faculty of Veterinary Medicine University of Tehran, 50(3&4), 1–5, [in Persian with English abstract].

[103] Nourian A. 1992 Serological study of toxoplasmosis in camels of Fars Province. DVM Dissertation, Shiraz University, [in Persian].

[104] Nourollahi Fard SR, Fathi S, Norouzi Asl E, Asgary Nazhad H, Salehzadeh Kazeroni S. 2012 Hard ticks on one-humped camel (*Camelus dromedarius*) and their seasonal population dynamics in southeast, Iran. Tropical Animal Health and Production, 44(1), 197–200. 21720786 10.1007/s11250-011-9909-y

[105] Nourollahi Fard SR, Ghalekhani N, Kheirandish R, Fathi S, Norouzi Asl E. 2012 The prevalence of *Linguatula serrata* nymphs in camels slaughtered in Mashhad slaughterhouse, Northeast, Iran. Asian Pacific Journal of Tropical Biomedicine, 2(11), 885–888. 23569865 10.1016/S2221-1691(12)60247-0 PMC3609234

[106] Nourollahi Fard SR, Kheirandish R, Fathi S, Norouzi Asl E. 2011 Prevalence of *Dipetalonema evansi* infection in *Camelus dromedaries*. Online Journal of Veterinary Research, 15(3), 261–269.

[107] Nyindo M, Lukambagire A-H. 2015 Fascioliasis: an ongoing zoonotic trematode infection. BioMed Research International, 2015, 786195. 26417603 10.1155/2015/786195 PMC4568335

[108] Oryan A, Khordadmehr M, Ranjbar VR. 2011 Prevalence, biology, pathology, and public health importance of linguatulosis of camel in Iran. Tropical Animal Health and Production, 43(6), 1225–1231. 21442154 10.1007/s11250-011-9830-4

[109] Oryan A, Moghaddar N, Hanifepour MR. 1993 Arthropods recovered from the visceral organs of camel with special reference to their incidence and pathogenesis in Fars Province of Iran. Indian Journal of Animal Sciences, 63(3), 290–293.

[110] Oryan A, Valinezhad A, Bahrami S. 2008 Prevalence and pathology of camel filariasis in Iran. Parasitology Research, 103(5), 1125–1131. 18629537 10.1007/s00436-008-1104-3

[111] Oryan A, Valinezhad A, Moraveji M. 2008 Prevalence and pathology of camel nasal myiasis in eastern areas of Iran. Tropical Biomedicine, 25(1), 30–36. 18600202

[112] Otranto D, Eberhard ML. 2011 Zoonotic helminths affecting the human eye. Parasites & Vectors, 4(1), 41. 21429191 10.1186/1756-3305-4-41 PMC3071329

[113] Otranto D, Giannelli A, Scotty Trumble N, Chavkin M, Kennard G, Latrofa MS, Bowman DD, Dantas-Torres F, Eberhard ML. 2015 Clinical case presentation and a review of the literature of canine onchocercosis by *Onchocerca lupi* in the United States. Parasites & Vectors, 8(1), 89. 25884672 10.1186/s13071-015-0699-3 PMC4346121

[114] Pasalary M, Arbabi M, Pashei S, Abdigoudarzi M. 2017 Fauna of ticks (Acari: Ixodidae) and their seasonal infestation rate on *Camelus dromedarius* (Mammalia: Camelidae) in Masileh region, Qom province, Iran. Persian Journal of Acarology, 6(1), 31–37.

[115] Pirali Kheirabadi K, Dehghani Samani A, Rajabi Vardanjani H. 2014 A report on the genital myiasis by *Wohlfahrtia magnifica* in camel herds in southwest of Iran. Veterinary Research Forum, 5(4), 329–332. 25610587 PMC4300001

[116] Pourjafar M, Azizi H, Darabi S, Khosravi M. 2007 The prevalence of nymphal stage of *Linguatula serrata* in camels (*Camelus dromedarius*) in Najaf-Abad. Journal of Camel Practice and Research, 14(2), 171–173.

[117] Pourjafar M, Badiei K, Sharifiyazdi H, Chalmeh A, Naghib M, Babazadeh M, Mootabi Alavi A, Hosseini Joshani-Zadeh N. 2013 Genetic characterization and phylogenetic analysis of *Trypanosoma evansi* in Iranian dromedary camels. Parasitology Research, 112(2), 899–903. 23007725 10.1007/s00436-012-3121-5

[118] Qablan MA, Sloboda M, Jirků M, Oborník M, Dwairi S, Amr ZS, Hořín P, Lukeš J, Modrý D. 2012 Quest for the piroplasms in camels: identification of *Theileria equi* and *Babesia caballi* in Jordanian dromedaries by PCR. Veterinary Parasitology, 186(3–4), 456–460. 22186193 10.1016/j.vetpar.2011.11.070

[119] Radfar MH, Aminzadeh Gowhari M. 2013 Common gastrointestinal parasites of indigenous camels (*Camelus dromedarius*) with traditional husbandry management (free-ranging system) in central deserts of Iran. Journal of Parasitic Diseases, 37(2), 225–230. 24431575 10.1007/s12639-012-0170-8 PMC3793086

[120] Radfar MH, Ebrahimy Maimand A, Sharify A. 2006 A report on parasitic infections in camel (*Camelus dromedarius*) of Kerman slaughterhouse. Journal of Faculty of Veterinary Medicine University of Tehran, 61(2), 165–168, [in Persian with English abstract].

[121] Radfar MH, Fathi S, Norouzi Asl E. 2010 Prevalence of *Linguatula serrata* nymphs in one-humped camel (*Camelus dromedaries*) in southeast of Iran. Scientia Parasitologica, 11(4), 199–202.

[122] Radfar MH, Gowhari MA, Khalili M. 2013 Comparison of capture ELISA and modified Ziehl-Neelsen for detection of *Cryptosporidium parvum* in feces of camel (*Camelus dromedarius*) in Iran. Scientia Parasitologica, 14(3), 147–152.

[123] Rafyi A, Maghami G. 1951 Action préventive et curative du méthylsulfate d’antrycide dans la trypanomiase à *T. evansi*. Archives of Razi Institute, 8(1), 59–63.

[124] Rahbari S, Bazargani TT. 1995 Blood parasites in camels of Iran. Journal of Veterinary Parasitology, 9, 45–46.

[125] Rahbari S, Bazargani TT, Rak H. 1981 Sarcocystosis in the camel in Iran. Journal of Faculty of Veterinary Medicine University of Tehran, 37, 1–10, [in Persian with English abstract].

[126] Rak H, Anwar M. 1974 Some diptera larvae causing myiasis in Iran. Entomologist’s Monthly Magazine, 110, 79–80.

[127] Rak H, Rahgozar R 1975 Demodectic mange in the eyelid of domestic ruminants in Iran. Bulletin de la Société de Pathologie Exotique et de ses Filiales, 68(6), 591–593. 1243891

[128] Ranjbar Bahadori S. 2003 Study of species diversity of animal ticks in Garmsar. Journal of Faculty of Veterinary Medicine University of Tehran, 58(1), 11–14, [in Persian with English abstract].

[129] Ranjbar Bahadori S, Afshari Moghaddam A. 2009 Study of the prevalence of haemoparasites in camels of Zabol County in the year 2008. Veterinary Clinical Pathology, 3(2), 503–507, [in Persian].

[130] Raoofi A, Kazempoor R, Akbarinejad V, Shojaei M, Tabatabaei SS. 2009 Natural trypanosomosis in a bactrian camel (*Camelus bactrianus*) in Iran. Journal of Camel Practice and Research, 16(2), 233–235.

[131] Razavi SM, Oryan A, Bahrami S, Mohammadalipour A, Gowhari M. 2009 Prevalence of *Cryptosporidium* infection in camels (*Camelus dromedarius*) in a slaughterhouse in Iran. Tropical Biomedicine, 26(3), 267–273. 20237440

[132] Razi Jalali MH, Dehghan S, Haji A, Ebrahimi M. 2016 Myiasis caused by *Cephalopina titillator* (Diptera: Oestridae) in camels (*Camelus dromedarius*) of semi-arid areas in Iran: distribution and associated risk factors. Comparative Clinical Pathology, 25(4), 677–680.

[133] Rezaei F, Tavassoli M, Javdani M. 2012 Prevalence and morphological characterizations of *Linguatula serrata* nymphs in camels in Isfahan Province, Iran. Veterinary Research Forum, 3(1), 61–65. 25653748 PMC4312821

[134] Robertson LJ, Björkman C, Axén C, Fayer R. 2014 Cryptosporidiosis in farmed animals, in *Cryptosporidium*: Parasite and Disease, Cacciò SM. Windmer G, Editors Springer: Vienna p. 149–235.

[135] Rocky A, Razi Jalali MH, Hajikolaei MRH, Hamidinejat H, Shirazi M. 2011 First report of infection of water buffalo with *Trypanosoma evansi* in Ahvaz city. in 2nd Iranian Congress of Veterinary Pathobiology Garmsar, Iran, [in Persian].

[136] Rokni MB. 2009 Echinococcosis /hydatidosis in Iran. Iranian Journal of Parasitology, 4(2), 1–16.

[137] Sadrebazzaz A, Haddadzadeh H, Shayan P. 2006 Seroprevalence of *Neospora caninum* and *Toxoplasma gondii* in camels (*Camelus dromedarius*) in Mashhad, Iran. Parasitology Research, 98(6), 600–601. 16425066 10.1007/s00436-005-0118-3

[138] Safarpoor Dehkordi F, Haghighi Borujeni MR, Rahimi E, Abdizadeh R. 2013 Detection of *Toxoplasma gondii* in raw caprine, ovine, buffalo, bovine, and camel milk using cell cultivation, cat bioassay, capture ELISA, and PCR methods in Iran. Foodborne Pathogens and Disease, 10(2), 120–125. 23441913 10.1089/fpd.2012.1311

[139] Salim Abadi Y, Telmadarraiy Z, Vatandoost H, Chinikar S, Oshaghi M, Moradi M, Mirabzadeh Ardakan E, Hekmat S, Nasiri A. 2010 Hard ticks on domestic ruminants and their seasonal population dynamics in Yazd province, Iran. Iranian Journal of Arthropod-Borne Diseases, 4(1), 66–71. 22808391 PMC3385546

[140] Salimi Bejestani MR, Changizi E, Darvishi MM. 2016 Abnormal life cycle of *Hyalomma dromedarii* (Acari: Ixodidae) on single-humped camels in Semnan, North-East of Iran. Archives of Razi Institute, 71(3), 195–198.

[141] Sazmand A, Anvari Tafti MH, Hekmatimoghaddam S, Moobedi I. 2013 *Dipetalonema evansi* infection in camels of Iran’s central area. Pakistan Journal of Biological Sciences, 16(13), 647–650. 24505989 10.3923/pjbs.2013.647.650

[142] Sazmand A, Eigner B, Mirzaei M, Hekmatimoghaddam S, Harl J, Duscher GG, Fuehrer H-P, Joachim A. 2016 Molecular identification and phylogenetic analysis of *Dipetalonema evansi* (LEWIS, 1882) in camels (*Camelus dromedarius*) of Iran. Parasitology Research, 115(4), 1605–1610. 26750131 10.1007/s00436-015-4896-y

[143] Sazmand A, Eigner B, Mirzaei M, Hekmatimoghaddam S, Harl J, Duscher GG, Fuehrer H-P, Joachim A. 2016 Molecular identification of hemoprotozoan parasites in camels (*Camelus dromedarius*) of Iran. Iranian Journal of Parasitology, 11(4), 568–573. 28127369 PMC5251186

[144] Sazmand A, Hamidinejat H, Hekmatimoghaddam S, Asadollahi Z, Mirabdollahi S. 2012 *Eimeria* infection in camels (*Camelus dromedarius*) in Yazd Province, central Iran. Tropical Biomedicine, 29(1), 77–80. 22543606

[145] Sazmand A, Rasooli A, Nouri M, Hamidinejat H, Hekmatimoghaddam S. 2011 Serobiochemical alternations in subclinically affected dromedary camels with *Trypanosoma evansi* in Iran. Pakistan Veterinary Journal, 31(3), 223–226.

[146] Sazmand A, Rasooli A, Nouri M, Hamidinejat H, Hekmatimoghaddam S. 2012 Prevalence of *Cryptosporidium* spp. in camels and involved people in Yazd Province, Iran. Iranian Journal of Parasitology, 7(1), 80–84. 23133476 PMC3488825

[147] Sazmand A, Razi Jalali MH, Hekmatimoghaddam S, Asadollahi Z. 2013 Seroprevalence of hydatidosis in camels of Yazd Province, Iran. Journal of Veterinary Laboratory Research, 5(2), 121–128, [in Persian with English abstract].

[148] Sazmand A, Tavassoli M, Ebrahimnejad B, Kazemnia A, Asadollahi Z. 2014 PCR assays for detection of *Toxoplasma gondii* infection in Iranian camels (*Camelus dromedarius*) of Yazd Province. Journal of Veterinary Clinical Pathology, 8(29), 383–389, [in Persian with English abstract].

[149] Schuster FL, Ramirez-Avila L. 2008 Current world status of *Balantidium coli*. Clinical Microbiology Reviews, 21(4), 626–638. 18854484 10.1128/CMR.00021-08 PMC2570149

[150] Serrano-Martínez E, Collantes-Fernández E, Chávez-Velásquez A, Rodríguez-Bertos A, Casas-Astos E, Risco-Castillo V, Rosadio-Alcantara R, Ortega-Mora LM. 2007 Evaluation of *Neospora caninum* and *Toxoplasma gondii* infections in alpaca (*Vicugna pacos*) and llama (*Lama glama*) aborted foetuses from Peru. Veterinary Parasitology, 150(1–2), 39–45. 17931783 10.1016/j.vetpar.2007.08.048

[151] Shahraki F. 2016 Prevalence of *Cryptosporidium parvum* in camels of Sistan region by ELISA and assessment of risk factors season, age and sex. DVM Dissertation, University of Zabol, [in Persian].

[152] Shakerian A, Hosseini SR, Abbasi M. 2011 Prevalence of *Cephalopina titillator* (Diptera: Oestridae) larvae in one-humped camel (*Camelus dromedarius*) in Najaf-Abad, Iran. Global Veterinaria, 6(3), 320–323.

[153] Shakerian A, Shekarforoush SS, Ghafari Rad H. 2008 Prevalence of *Linguatula serrata* nymphs in one-humped camel (*Camelus dromedarius*) in Najaf-Abad, Iran. Research in Veterinary Science, 84(2), 243–245. 17582449 10.1016/j.rvsc.2007.04.015

[154] Shaki M, Tājbaḵš Ḥ, Sajjādī Ṣ. 1993 Dām Pezeškī (veterinary medicine), Encyclopaedia Iranica, VI/6; available online at http://www.iranicaonline.org/articles/dam-pezeski-veterinary-medicine (accessed online at 2 June 2017)..

[155] Sharafi SM, Rostami-Nejad M, Moazeni M, Yousefi M, Saneie B, Hosseini-Safa A, Yousofi-Darani H. 2014 *Echinococcus granulosus* genotypes in Iran. Gastroenterology and Hepatology from Bed to Bench, 7(2), 82–88. 24834298 PMC4017566

[156] Sharbatkhori M, Fasihi-Harandi M, Mirhendi H, Hajialilo E, Kia EB. 2011 Sequence analysis of cox1 and nad1 genes in *Echinococcus granulosus* G3 genotype in camels (*Camelus dromedarius*) from central Iran. Parasitology Research, 108(3), 521–527. 20922418 10.1007/s00436-010-2092-7

[157] Sharbatkhori M, Mirhendi H, Jex AR, Pangasa A, Campbell BE, Kia EB, Eshraghian MR, Harandi MF, Gasser RB. 2009 Genetic categorization of *Echinococcus granulosus* from humans and herbivorous hosts in Iran using an integrated mutation scanning-phylogenetic approach. Electrophoresis, 30(15), 2648–2655. 19637222 10.1002/elps.200900145

[158] Sharbatkhori M, Tanzifi A, Rostami S, Rostami M, Fasihi Harandi M. 2016 *Echinococcus granulosus* sensu lato genotypes in domestic livestock and humans in Golestan Province, Iran. Revista do Instituto de Medicina Tropical de São Paulo, 58, 38. 27253740 10.1590/S1678-9946201658038 PMC4879995

[159] Shekarforoush SS, Shakerian A, Hasanpoor MM. 2006 Prevalence of *Sarcocystis* in slaughtered one-humped camels (*Camelus dromedarius*) in Iran. Tropical Animal Health and Production, 38(4), 301–303. 17137132 10.1007/s11250-006-4362-z

[160] Shimi A. 1940 Study of surra disease in Iran. DVM Dissertation, University of Tehran, [in Persian].

[161] Spotin A, Mahami-Oskouei M, Harandi MF, Baratchian M, Bordbar A, Ahmadpour E, Ebrahimi S. 2017 Genetic variability of *Echinococcus granulosus* complex in various geographical populations of Iran inferred by mitochondrial DNA sequences. Acta Tropica, 165, 10–16. 26948902 10.1016/j.actatropica.2016.03.002

[162] Swelum AA, Ismael AB, Khalaf AF, Abouheif MA. 2014 Clinical and laboratory findings associated with naturally occurring babesiosis in dromedary camels. Bulletin of the Veterinary Institute in Pulawy, 58(2), 229–233.

[163] Tadayon S, Sharifiyazdi H, Moazeni M, Divar MR. 2015 Molecular differentiation of *Fasciola* species and characterization of genetic diversity of *F. gigantica* using NADH dehydrogenase I (ND1) gene in the endemic areas of Iran. Iranian Journal of Parasitology, 10(1), 9–18. 25904941 PMC4403545

[164] Tadjbakhsh H. 1994 Traditional methods used for controlling animal diseases in Iran. Revue Scientifique et Technique (International Office of Epizootics), 13(2), 599–614. 8038455 10.20506/rst.13.2.777

[165] Tajik H, Tavassoli M, Khani H, Javadi S. 2007 Prevalence of *Linguatula serrata* nymphs in slaughtered camels of Iran. Journal of Camel Practice and Research, 14(1), 69–71.

[166] Tajik J, Moghaddar N, Nikjou D, Taleban Y. 2011 Occurrence of gastrointestinal helminths in Bactrian camel in Iran. Tropical Biomedicine, 28(2), 362–365. 22041757

[167] Tajik J, Nourollahi Fard SR, Paidar A, Anousheh S, Dehghani E. 2013 Balantidiasis in a dromedarian camel. Asian Pacific Journal of Tropical Disease, 3(5), 409–412.

[168] Tomassone L, Grego E, Callà G, Rodighiero P, Pressi G, Gebre S, Zeleke B, De Meneghi D. 2012 Ticks and tick-borne pathogens in livestock from nomadic herds in the Somali Region, Ethiopia. Experimental & Applied Acarology, 56(4), 391–401. 22349943 10.1007/s10493-012-9528-y

[169] Valinezhad A, Oryan A, Ahmadi N. 2008 *Sarcocystis* and its complications in camels (*Camelus dromedarius*) of eastern provinces of Iran. Korean Journal of Parasitology, 46(4), 229–234. 19127328 10.3347/kjp.2008.46.4.229 PMC2612607

[170] Vosooghi Afshar A. 1976 Study of occular *Thelazia* parasites in the eyes of domestic ruminants in Tehran slaughterhouse. DVM Dissertation, University of Tehran, [in Persian].

[171] Wernery U, Kaaden OR. 2002 Infectious Diseases in Camelids, 2nd edn Blackwell Science: Berlin.

[172] Wernery U, Kinne J, Schuster RK. 2014 Camelid Infectious Disorders. Paris: OIE (World Organisation for Animal Health).

[173] Yakhchali M, Athari S. 2010 A study on prevalence of *Eimeria* spp. infection in camels of Tabriz region. Archives of Razi Institute, 65(2), 111–115.

[174] Yakhchali M, Cheraghi E. 2007 Eimeriosis in Bactrian and dromedary camels in the Miandoab region, Iran. Acta Veterinaria (Belgrade), 57(5–6), 545–552.

[175] Yakhchali M, Moradi T. 2012 Prevalence of *Cryptosporidium*-like infection in one-humped camels (*Camelus dromedarius*) of northwestern Iran. Parasite, 19(1), 71–75. 22314242 10.1051/parasite/2012191071 PMC3671425

[176] Yazdanbakhsh A, Hatami H, Sharifiarab G, Arabameri M. 2016 Evaluation of slaughtered camels liver infection in Shahroud in 2015. Journal of Knowledge & Health, 11(3), 49–54, [in Persian with English abstract].

[177] Youssef SY, Yasien S, Mousa WMA, Nasr SM, El-Kelesh EAM, Mahran KM, Abd-El-Rahman AH. 2015 Vector identification and clinical, hematological, biochemical, and parasitological characteristics of camel (*Camelus dromedarius*) theileriosis in Egypt. Tropical Animal Health and Production, 47(4), 649–656. 25677167 10.1007/s11250-015-0771-1

[178] Zakian A, Nouri M, Safaei P, Mohammad-Sadegh M, Kahroba H, Mokhber Dezfouli MR, Moallemian R. 2017 An acute outbreak of natural *Trypanosoma evansi* infection in camel (*Camelus dromedarius*) herds in the southwestern Iran. Comparative Clinical Pathology, 26(1), 51–59.

[179] Zangooyi F. 2017 Molecular study of infection of camels of Sistan region to *Trypanosoma evansi*. DVM Dissertation, University of Zabol, [in Persian].

[180] Zarif Fard MR, Hashemi Fesharaki R. 2000 Study on tissue and blood protozoa of camels in southern Iran. Journal of Camel Practice and Research, 7(2), 193–194.

